# Surface Treatment Strategies and Their Impact on the Material Behavior and Interfacial Adhesion Strength of Shape Memory Alloy NiTi Wire Integrated in Glass Fiber-Reinforced Polymer Laminate Structures

**DOI:** 10.3390/ma17143513

**Published:** 2024-07-16

**Authors:** Saravanan Palaniyappan, Harshan Kalenahalli Ramesha, Maik Trautmann, Steven Quirin, Tobias Heib, Hans-Georg Herrmann, Guntram Wagner

**Affiliations:** 1 Group of Composites and Material Compounds (PVW), Institute of Materials Science and Engineering (IWW), Chemnitz University of Technology, 09125 Chemnitz, Germany; harshankrjc@gmail.com (H.K.R.); maik.trautmann@mb.tu-chemnitz.de (M.T.); guntram.wagner@mb.tu-chemnitz.de (G.W.); 2 Chair for Lightweight Systems, Saarland University, 66123 Saarbrücken, Germany; steven.quirin@uni-saarland.de (S.Q.); tobias.heib@uni-saarland.de (T.H.); hans-georg.herrmann@uni-saarland.de (H.-G.H.); 3 Fraunhofer Institute for Nondestructive Testing IZFP, Campus E3 1, 66123 Saarbrücken, Germany

**Keywords:** GFRP laminates, shape memory alloy, NiTi, surface treatments

## Abstract

Over the past few decades, there has been a growing trend in designing multifunctional materials and integrating various functions into a single component structure without defects. This research addresses the contemporary demand for integrating multiple functions seamlessly into thermoplastic laminate structures. Focusing on NiTi-based shape memory alloys (SMAs), renowned for their potential in introducing functionalities like strain measurement and shape change, this study explores diverse surface treatments for SMA wires. Techniques such as thermal oxidation, plasma treatment, chemical activation, silanization, and adhesion promoter coatings are investigated. The integration of NiTi SMA into Glass Fiber-Reinforced Polymer (GFRP) laminates is pursued to enable multifunctional properties. The primary objective is to evaluate the influence of these surface treatments on surface characteristics, including roughness, phase changes, and mechanical properties. Microstructural, analytical, and in situ mechanical characterizations are conducted on both raw and treated SMA wires. The subsequent incorporation of SMA wires after characterization into GFRP laminates, utilizing hot-press technology, allows for the determination of interfacial adhesion strength through pull-out tensile tests.

## 1. Introduction

The rise of smart materials and structures is revolutionizing design principles in structural engineering, robotics, and aerospace, where one can find a growing demand for lightweight and adaptable systems. Shape memory alloys (SMAs) are crucial smart materials that offer high actuation energy, significant actuation strain, and recovery stress [1] and are used in various fields and applications, namely, bone plates [2], self-expandable stents [3], and actuators in the automotive field [4]. SMAs are also suitable for composite materials and smart composite structures due to their shape memory effect (SME) and superelasticity [1]. The SME of SMAs involves deformation in the martensite phase at low temperatures and shape recovery in the austenite phase at high temperatures. When the recovery is restricted, SMAs experience substantial tensile stresses. Superelasticity enables the material to recover a significant amount of strain when deformed above the austenite finish temperature. Notably, the SME of SMAs generates actuation stress ranging from 100 to 700 MPa, surpassing the actuation stresses produced by low-power hydraulic actuators (20 to 70 MPa) and piezoelectric actuators (1 to 9 MPa) [5]. NiTi-based SMA is widely considered the preferred commercial choice among smart materials. This preference is due to several advantages, when compared to other types of SMAs, such as iron-based and copper-based SMAs, including lower production costs, easier and safer handling, and superior mechanical properties [4]. It could also be incorporated in small filigree specimens in the future, additively manufactured by laser powder bed fusion [6]. Although NiTi has potential applications in many different fields, integrating it in a polymer composite is a complex process that requires an understanding of different fundamental aspects. It is feasible to manufacture quite large components; however, complex shapes are still limited. Different methodologies have been proposed for the optimization of the structure and as to where the stiffer elements are to be positioned [7]. Furthermore, to fully utilize the advantages of SMA, it is essential to establish a strong bond interface between the SMA wire and the surrounding matrix. This bond interface enables the efficient transfer of stress from the pre-strained SMA wire to the matrix during the phase transformation process. The interfacial strength should be sufficiently high under normal conditions. However, it should not be excessively stiff to the point where cracks propagate rapidly, leading to undetectable progressive damage and potentially catastrophic failure [8]. From different metal–polymer systems, the influence of the surface roughness of the metal and joining process temperature for the composite is already known to be notable [9].

Multiple studies have delved into different strategies to optimize the interface between NiTi SMAs and polymer matrices, with a common goal of improving the adhesive bond and overall material performance. Mazharul et al. investigated the influence of pre-strain and temperature on the adhesion strength of NiTi SMFs embedded in epoxy adhesives [10]. Their findings revealed that increasing pre-strain positively affected the interfacial shear strength (IFSS), attributed to enhanced surface roughness and improved interaction between the SMFs and the adhesive. Similarly, Senhal et al. utilized laser engraving to create channels on NiTi surfaces, resulting in enhanced adhesion of the polymer matrix [11,12]. While these approaches showed improvements in adhesion strength, they also highlighted the trade-off between adhesion enhancement and the potential reduction in shape-memory properties of the composite material, accounting for the residual transformation strain accumulated during the treatment conditions.

Chemical treatments and coatings have also been explored to enhance adhesion strength [13,14]. Bin Yang et al. employed acid treatment and nano-silica particle coating on NiTi wires, resulting in a notable increase in interfacial adhesion strength [13]. Li-min et al. (2014) utilized electrochemical deposition of zinc oxide (ZnO) nanostructures on NiTi wires, creating nail-like structures that significantly improved adhesion with the epoxy matrix [14]. These studies underscore the effectiveness of chemical modifications in enhancing adhesion strength, while also highlighting the importance of understanding the underlying mechanisms governing the interaction between modified surfaces and polymer matrices. Mechanical methods, such as wire twisting and indentation, have been investigated to improve adhesion strength [8,15]. Lau et al. demonstrated that wire twisting effectively increased surface roughness and binding between NiTi wires and epoxy matrices [15]. Similarly, Yuan et al. proposed a mechanical indentation method, resulting in a significant enhancement in interfacial adhesion strength compared to conventional surface treatments [8]. These mechanical approaches in comparison to other surface treatment strategies offer promising avenues for enhancing adhesion strength while maintaining the structural integrity of the composite material. Looking ahead, there is a need to explore novel approaches and alternative polymer matrices to further optimize the performance of NiTi–polymer composite materials [16,17], as most studies have focused on epoxy resin matrices [18,19]. Through a comprehensive understanding of surface modification techniques, chemical treatments, and mechanical methods, researchers can continue to advance the development of high-performance NiTi-based composite materials with tailored properties for various applications.

This research study explores five methods to enhance the interfacial bonding between NiTi wires and a polyamide matrix in GFRP laminates. It aims to enhance the compatibility of NiTi with polyamide by using thermal treatment, plasma treatment, surface activation, silanization, and adhesion-promoting coating. Thermal and plasma treatments create surface roughness and optimize oxide thickness on NiTi, facilitating mechanical interlocking with the polyamide matrix. Surface activation and silanization promote hydroxide and covalent bonding, respectively. Additionally, a commercial adhesion promoter is applied to examine its combined effect with surface activation. NiTi-integrated polyamide laminates were manufactured via hot-press molding. Characterization methods include single-wire tensile tests, XRD, LSM, SEM, and single-wire pull-out tests. Overall, the study aims to analyze the impact of various surface treatments on the material properties and interfacial adhesion strength of Ni wire integrated with GFRP laminates.

## 2. Materials and Methods

### 2.1. Materials

Nickel–titanium wires (Fort Wayne Metals Limited, Castlebar, Ireland) with a diameter of 250 μm were used in this study. The manufactured wires were straight annealed providing a light oxide finish. Before every surface treatment procedure, the NiTi samples underwent thorough cleaning for 15 min each, utilizing ultrasonication in ethanol, and DI water, to meticulously remove contaminants such as grease, dust, foreign particles, etc.

For surface activation experiments, Perhydrol™, 30% hydrogen peroxide solution (H_2_O_2_), and 25% ammonium hydroxide (NH_4_OH) were sourced from Sigma Aldrich Darmstadt, Germany. For pickling after surface activation, sodium hydroxide (NaOH) was sourced from VWR Chemicals, Germany. For silanization experiments, 3-Glycidoxypropyltrimethoxysilane (3-GPTMS) (abcr GmbH, Karlsruhe, Germany) and Xylene (TH. Geyer, Renningen, Germany) were used.

VESTAMELT^®^ Hylink copolyamide adhesion promoter was sourced from Evonik Industries, Herne, Germany. To produce GFRP laminates, unidirectional glass fiber-reinforced Polyamide 6 prepregs (GFPA6 4/1) with a fiber volume content of 50% (Cetex Institut gGmbH, Chemnitz, Germany) were used. The glass fiber prepregs were oven-dried at 60 °C for 24 h to remove any moisture before using them in the hot-press lamination process.

A summary of the materials used in accordance with each of the pre-defined surface treatment methodologies is listed in Table 1.

### 2.2. Surface Treatment Methodologies on NiTi Wires

#### 2.2.1. Thermal Treatments

NiTi wire surfaces underwent thermal oxidation in a TF600 tube furnace (Carbolite Gero GmbH & Co. KG, Neuhausen, Germany). Initially, 250 µm NiTi wires were cut into 150 mm lengths and cleaned through ultrasonication in ethanol, and DI water for 15 min each. The cleaned wires were then positioned in a quartz tube and subjected to a temperature ramp of 10 °C/min, oxidizing at 400 °C (TO400), 500 °C (TO500), and 600 °C (TO600) for 30 min each under argon gas flow. Throughout the heating ramp, the samples were shielded from oxidation by maintaining a flow of argon gas. Once the desired temperature was attained, the argon flow was replaced by airflow. After treatment, samples were cooled naturally and stored in capped tubes originally for nuclear magnetic resonance spectroscopy (NMR) to prevent further oxidation.

#### 2.2.2. Plasma Treatments

Plasma treatment (PT) and plasma-induced oxidation (PO) were conducted using the CVD device (FHR Anlagenbau GmbH, Ottendorf-Okrilla, Germany). NiTi wire samples, cut and cleaned beforehand, were securely positioned on a sample holder at the center of a specialized reactor quartz tube. An inductive HF plasma, powered at 500 W at a working pressure of 0.6 mbar was used with a controlled flow of 100 sccm argon for PT and a gas mixture of 70 sccm argon and 30 sccm Oxygen for PO. The treatment process duration was 120 min, during which the chamber edge temperature peaked at 380 °C within the initial 20 min. After treatment, samples were delicately removed from the tube and stored in NMR glass tubes to maintain treatment effects. The PT aimed at bombarding argon ions onto the NiTi wire surface, causing physical etching of contaminants and roughening the surface to increase the sample surface area. PO aimed at exploring the impact on surface oxidation by directing argon and oxygen ions toward the NiTi wire surface, resulting in the physical etching of contaminants and the formation of oxides through reactive oxygen species.

#### 2.2.3. Chemical Treatments and Coatings

The NiTi wires were subjected to controlled oxidation in a base piranha solution at 60 °C for 20 min. This solution, composed of a 7:1 volumetric ratio of 30% concentrated ammonium hydroxide and 30% hydrogen peroxide, induced oxidation. After oxidation, the wires underwent chemical treatment and pickling in a sodium hydroxide solution for 15 min to boost surface hydroxide concentration. This pickling process was repeated three times to ensure optimal surface activation. Following this, the surface-activated samples were thoroughly rinsed in deionized water before being dried with a blower. Finally, the treated wires were carefully stored in NMR tubes.

Once surface-activated, the NiTi wires were immediately utilized in a coating process involving organosilane. In a bath, 12 g of 3-glycidoxypropyltrimethoxysilane (GPTMS) was mixed with 300 mL of xylene and heated to 110 °C to initiate the chemical reaction. The treated NiTi wires were then immersed in the silane solution bath. During this stage, the methoxy group in GPTMS reacted with the surface hydroxides, forming silicone–oxygen bonds with the NiTi wire surface. To ensure a complete reaction, the wires remained in the silane solution under a magnetic stirrer for 20 min. After the reaction was completed, the wires were removed from the silane bath and cleaned using ethanol and deionized water to eliminate any loosely attached GPTMS. Finally, the samples were dried with a blower and stored in NMR tubes.

To further improvise the surface characteristics, NiTi wires were prepared for powder coating using the GEMA Optiflex 2S system with the GEMA PG1 spray gun. Surface-activated (SA) NiTi wires were coated with an adhesion promoter (AP) to assess combined treatment effects. The adhesion promoter, VESTAMELT^®^ Hylink, was sprayed onto the wires with a 100 kV electrostatic charge at 0.7 bar pressure. Coated wires with a coating thickness of approx. 30–50 µm were cured in an oven at 200 °C for 5 min for proper adhesion. This method offers a systematic approach for effective powder coating on NiTi wires, and the wires were stored in NMR tubes. The AP is used as an additional methodology to check for better adhesion properties and not as the main surface treatment. The SEM and roughness characterizations were excluded because the 30–50 µm coating mostly shows the surface properties of the co-polyamide layer and not the NiTi wires.

### 2.3. Hot-Press Molding

The hot-press lamination procedure for treated NiTi wires was conducted using the Plattenpress P 300 P/M machine manufactured by Dr. Collin GmbH (Maitenbeth, Germany). Employing a mold measuring 60 × 60 mm, glass fiber polyamide prepreg was tailored to match its dimensions. NiTi wires, totaling 60 mm in length, were positioned in a 0° orientation between the lower and upper layers of glass fiber polyamide prepreg. Approximately 20 mm of the NiTi wires were situated on the GF prepreg, leaving the remaining 40 mm extending outward.

To ensure proper alignment and reinforcement during molding, the uppermost two and lowermost two plies of glass fiber polyamide prepreg were oriented at a 90° angle to the NiTi wires. Precise control was maintained over the lamination process parameters, encompassing temperature, duration, and pressure. Following molding, the specimens were cooled, extracted from the mold, and meticulously trimmed to eliminate any excess material. Subsequently, they were cut into the requisite shape and size for the measurement of single-fiber pullout strength.

### 2.4. Characterization Techniques

#### 2.4.1. Tensile Testing of Wires

To examine in situ resistance changes during tensile deformation, single-wire tensile tests were conducted utilizing a Zwick line 500 N tensile testing machine. The test design closely followed ASTM F2516 standards [20]. NiTi wires, with a gauge length of 20 mm, were prepared for the tests. A preload of 1N was applied, and the test speed was maintained at 0.5 mm/min. All measurements were conducted at room temperature, and in situ resistance measurements were simultaneously performed using the HIOKI RM3545 Resistance meter (HIOKI Europe GmbH, 65760 Eschborn, Germany).

#### 2.4.2. Surface Analyses of Wires

To determine the nominal surface properties like the average surface roughness (S_a_), skewness (R_sk_), and kurtosis (R_ku_) of the surface of treated and non-treated NiTi wires, a laser scanning microscope (LSM—Keyence VKX-200K) has been used. On each sample type, an area of 125 × 300 µm on the surface of the NiTi wires has been captured using a 50× objective, and the curvature has been flattened to analyze with the inbuilt analysis software from Keyence (Osaka, Japan). The measurements were made on 30 different positions on three different samples of each type (10 measurement positions per sample). While the S_a_ values provide comprehensive information on the surface features of the treated and non-treated wires, the skewness and kurtosis of the surface provide more comprehensive statistical attributes of the surface.

In addition, a scanning electron microscope (SEM—Carl Zeiss, NEON 40EsB) has been employed to analyze the fracture sections and surface topography of treated and non-treated wires.

#### 2.4.3. Wire Pull-Out Tests

To investigate the interfacial bonding strength (shear strength) between the embedded NiTi wires and the surrounding GFRP laminates, wire pull-out tests from the hot-pressed and tailored laminates were conducted in correlation to DIN SPEC 19289:2022-08 [21]. Samples with the dimension of 15 × 30 mm containing a total of 60 mm NiTi wires with 20 mm wires embedded in the laminate structure and the remaining 40 mm as free-standing wire were cut out from the hot-pressed laminates. The pull-out tests were conducted using a Zwick line 500 N tensile testing machine (ZwickRoell, Ulm, Germany) at an elongation speed of 0.1 mm/s. The test continued until the NiTi wire was pulled out until the force dropped under 50% of the maximum force (F_max_). The interfacial bonding strength between the NiTi wire and the GFRP matrix was calculated using the equation below.

(1) $$\tau =\frac{F_{max}}{\pi DL}$$

where *τ* is the interfacial adhesion strength, *F_max_* is the maximum force applied, *D* is the diameter of the embedded NiTi wire, and *L* is the embedded length of the NiTi wire.

#### 2.4.4. XRD Analyses

X-ray diffraction analyses on treated and non-treated NiTi wires were conducted to analyze their composition and structure using a D8 Discover instrument (Bruker, Karlsruhe, Germany), employing Co-Kα radiation with a point focus, a 0.5 mm collimator, and a LynxEye XE-T detector. The measurement time was approximately 12 h.

## 3. Results and Discussions

### 3.1. Surface Topography Characterization

#### 3.1.1. SEM Analyses

**Figure 1:**
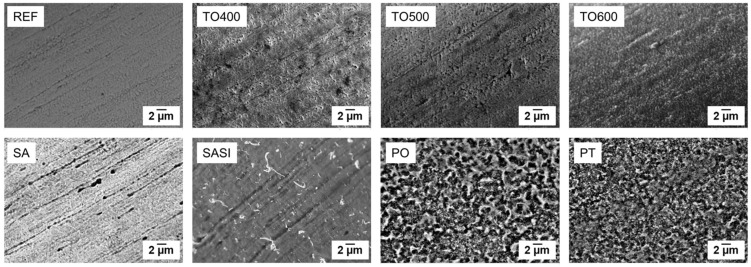
The surface topology of the non-treated reference and treated NiTi wires characterized using SEM.

SEM images in Figure 1 depict the surface topography of untreated and treated wires.

The REF wires displayed flawlessly processed, porous surfaces with distinct extrusion lines. After oxidation at 400 °C (TO400), some changes in pore size were noted, but extrusion lines remained visible, and no surface oxidation signs were apparent. At 500 °C (TO500), wire surface morphology resembled that of TO400, with larger pores and diminishing extrusion lines, indicating potential high-temperature oxidation effects. Furthermore, pores began forming a network of cracks, altering the wire’s microstructure. At 600 °C (TO600), a notable transformation occurred, with no discernible porous features but a uniform, coarse oxide layer across the wire, suggesting significant oxidation. The disappearance of extrusion lines indicated their substantial alteration due to high-temperature oxidation, which is potentially able to impact mechanical properties and structural integrity.

Plasma-treated wires (PT) exhibited thick oxide layers and evident cracks (in Figure 1), suggesting significant surface morphology alterations and mechanical deformations. The rough texture from plasma etching indicated material erosion, affecting wire microstructure and properties. Plasma-oxidized wires (PO) displayed similar features, with pronounced surface etching due to oxygen ion action, potentially influencing mechanical properties and interactions. SEM analysis of Piranha solution-etched wires (SA) showed reduced surface porosity compared to references, with extrusion line marks persisting, indicating partial retention of original features. The thin oxide grains observed likely resulted from Piranha solution etching. Silane-coated wires (SASI) exhibited a thin, undetermined silane layer on the surface.

#### 3.1.2. LSM Analyses

**Figure 2:**
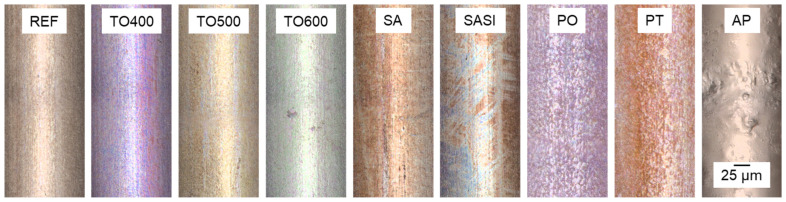
Surface topology of non-treated reference and treated NiTi wires characterized using LSM.

The LSM investigation delves into the surface roughness of both treated and untreated NiTi wires. The surface topology of those wires as pictured by LSM are given in Figure 2. The LSM images show visual differences between each of the treated wires’ surface topology, representing the nature of oxide formation via thermal treatments and a rougher texture creation via plasma treatments.

Roughness measurements were meticulously obtained from 30 different positions across various samples of each of the surface treatment variants. Employing box plots, the data are statistically analyzed, offering perspectives on the roughness distribution.

**Figure 3:**
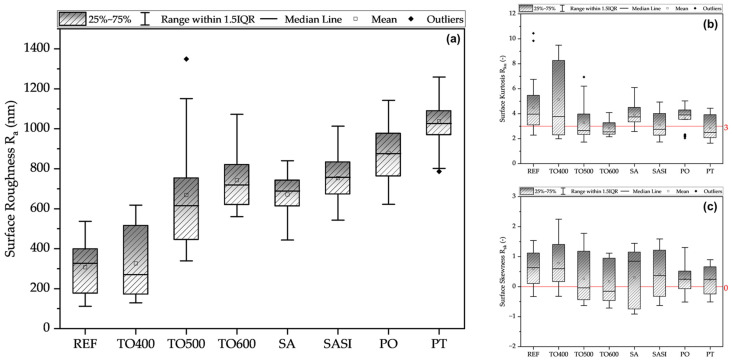
Representation of the variation of (**a**) surface roughness, (**b**) surface kurtosis, and (**c**) surface skewness of treated NiTi wires in comparison to non-treated reference wires.

The box plots of surface roughness variation in addition to the statistical surface kurtosis and skewness characteristics are shown in Figure 3. The observed surface roughness trends, as discussed previously in correlation with SEM results, can be attributed to alterations in surface morphology induced by various treatments. For instance, the REF had an average R_a_ of 305 nm, with SEM showing a smooth surface. With TO400 and TO500, the roughness increased from 325 nm to 666 nm, likely due to initial oxide and oxide spot formation, respectively. The highest oxidation temperature of 600 °C (TO600) correlated with further increased roughness (750 nm), consistent with SEM observations. Surface treatments SA and SASI showed similar roughness, aligning with SEM observations. Conversely, PO had higher roughness (880 nm), in line with SEM images showing dense etched and oxidized layers. The PT exhibited the highest roughness (1000 nm), possibly due to thick etched and oxide layers, as observed in SEM.

Skewness values indicated an abundance of peaks on wire surfaces. Kurtosis values reflected peak sharpness, with values below three suggesting flatter surfaces and values above three suggesting sharper peaks. REF and TO400 showed kurtosis values above three, suggesting sharp peaks. Higher oxidation temperatures led to values approaching three, indicating balanced surface profiles. TO600 and PT had values below three, suggesting flatter surfaces due to thin oxide layers. SA and PO showed values above three, indicating relatively sharper peaks, likely due to surface activation and plasma oxidation processes. SASI exhibited an intermediate value of around three.

### 3.2. Material Characterization

#### XRD

**Figure 4:**
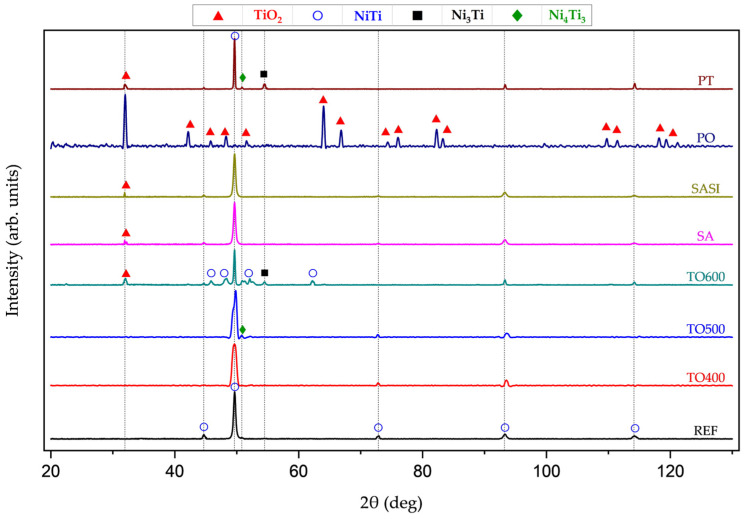
XRD plots of treated and non-treated reference NiTi wires.

The X-ray diffractogram of all the treated and non-treated wires as shown in Figure 4, were acquired to identify the phase transformations and crystal structure of the NiTi wires after surface treatments. The peaks observed in the non-treated REF NiTi wire at 2θ angles of 49.6°, 72.8°, 93.2°, and 114.1° correspond to crystallographic planes (hkl) of (110), (200), (211), and (220), respectively. These peaks confirm the body-centered cubic (BCC) (B2) crystal structure of the NiTi wire, with lattice constants a = b = c = 3.015, indicating the parent austenite phase.

At 400 °C (TO400), XRD analysis of thermally oxidized samples did not reveal any additional peaks, indicating an exceptionally thin oxidation layer, likely a few nanometers thick, undetectable by the device. At 500 °C (TO500), no distinctive oxide peaks were observed. However, a small peak at the 2θ angle of 50.7 °, indexed to crystallographic planes (hkl) of (212), suggests a transformation in crystalline structure from equiatomic NiTi to Ni_4_Ti_3_. This transformation indicates a chemical reaction of radially reactive titanium atoms, resulting in a decreased titanium atomic percentage in the alloy [22]. Analysis of samples treated at 600 °C (TO600) unveiled a peak at the 2θ angle of 31.7°, corresponding to the (110) plane of tetragonal anatase TiO_2_, and another peak at 54.6°, corresponding to the (202) plane of hexagonal Ni3Ti. This suggests a transformation of the material’s composition, with titanium possibly converting into TiO_2_ and hexagonal Ni_3_Ti phases. Furthermore, peaks observed at 45.7° (101 plane), 48.0° (111 plane), and 52.9° (111 plane) indicate the presence of monoclinic NiTi (B19′) as an intermediate phase during the transformation of NiTi.

PT wires revealed a distinct peak at 31.7°, indicating the presence of anatase TiO_2_ with tetragonal crystalline structure. Additionally, a notable peak at 54.6° was observed, corresponding to the (202) crystallographic plane of hexagonal Ni_3_Ti, indicating the formation of this phase within the material [23]. In the case of PO samples, a prominent peak at 49.6° was absent, indicating the formation of a dense oxide layer, typical of oxidation, which masks the NiTi crystallographic plane (110).

Distinct peaks corresponding to anatase TiO_2_ are identified at 31.7° (110), 47.9° (111), 66.3° (220), and 75.4° (310), along with peaks associated with rutile TiO_2_ at 41.8° (101), 45.5° (200), and 63.5° (211). These findings collectively suggest the formation of both anatase and rutile phases of TiO_2_ on the material’s surface post-plasma oxidation. The absence of the NiTi peak, alongside strong TiO_2_ peaks, signifies significant surface chemical and structural changes due to oxidation, highlighting the efficacy of plasma oxidation in inducing substantial surface modifications and the development of titanium oxide phases [24].

XRD analyses revealed a prominent peak at 31.7° in both surface-activated (SA) and silane-coated (SASI) samples, corresponding to the (110) plane indicative of anatase TiO_2_. This suggests oxidation of the NiTi alloy, particularly affecting titanium. Notably, titanium oxides, like anatase TiO_2_, were predominantly formed over nickel oxides, contrary to expectations. This preferential oxidation of titanium may stem from its higher reactivity with oxidizing agents in the piranha solution, leading to the observed anatase TiO_2_ peak [25].

### 3.3. Mechanical Characterization

#### 3.3.1. Tensile Testing Results

**Figure 5:**
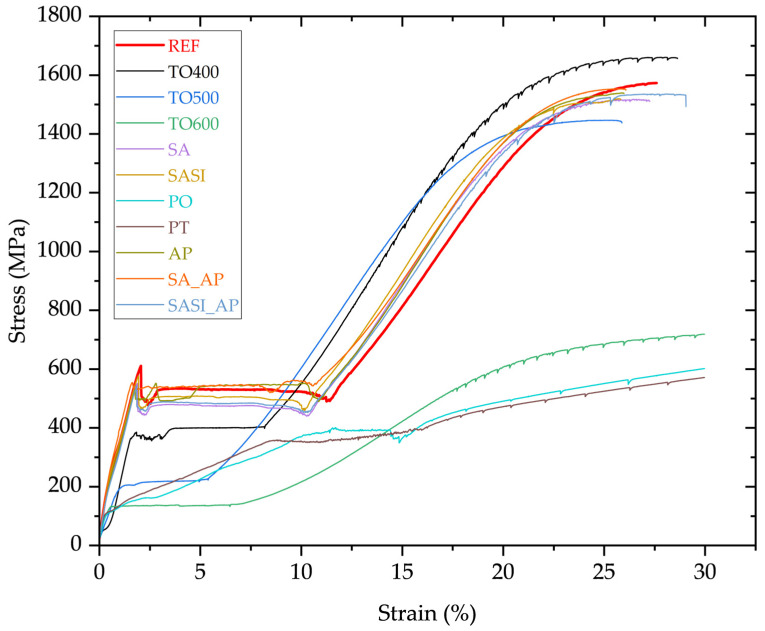
Stress vs. strain relationship of non-treated reference and treated NiTi wires at room temperature.

The stress–strain relationship of non-treated and treated NiTi wires is graphically represented in Figure 5.

Taking a closer look at the non-treated reference curve, a transition from the initial austenite (B2) phase to a stress-induced martensite phase (B19’) occurs at an applied stress of approximately 600 MPa. During this phase transition, the stress remains nearly constant despite the increasing strain, and this is the characteristic superelastic behavior of NiTi materials. This allows the material to undergo significant reversible deformation and is called the plateau region with the capacity to absorb and dissipate energy efficiently. This transition, however, is not a direct one. It is accompanied by the appearance of a rhombohedral R-phase, characterized by a strain of about 0.9%. This introduction of the R-phase coincides with a reduction in stress to around 500 MPa, within a range of ±20 MPa. During this phase transition, there is a change in the crystal structure of the material [22]. Throughout the mechanical deformation process, twinned martensite transforms into detwinned martensite. This process, known as martensite reorientation, contributes to the overall behavior observed during deformation [23]. At approximately 8% elongation, the martensitic transformation becomes complete. Beyond this point, the NiTi material undergoes plastic deformation, leading to a gradual increase in stress. Eventually, the wire fractures at an elongation of roughly above 25%.

Thermal oxidation of NiTi wires at 400 °C, 500 °C, and 600 °C (TO400, TO500, and TO600) brings about significant changes in both their mechanical and electrical properties. In the case of TO400, the stress-induced martensite start (M_s_), indicating the transition from the B2 to B19′ phase, occurs at approximately 400 MPa with a margin of ±10 MPa. Additionally, there is a distinct observation of an R-phase, showing a strain of 0.7% under a stress of 400 MPa, which differs from the behavior of untreated NiTi wires (where this occurs at around 600 MPa). Interestingly, the TO400 wires do not exhibit the typical stress drop associated with the R-phase transition, suggesting a unique reaction, and the decrease in M_s_ stress is linked to thermal effects, likely caused by the relaxation of internal stress [24]. Furthermore, there is a reduction in the plateau of pseudo-elasticity from 8% to 6% due to phase alterations induced by thermal oxidation, as the B2 structure transforms into a likely monoclinic B19′ phase due to changes in surface composition. In the case of TO500, M_s_ initiates at around 200 MPa, with a tolerance of ±10 MPa, and the pseudo-elasticity region diminishes to approx. 4%. This is also attributed to the same phase transformation principles noticed in TO400 and XRD investigations revealed the presence of Ni_3_Ti, a monoclinic crystal structure akin to the B19′ phase. In the case of TO600, the R-phase is notably absent, and this clearly portrays that the wires are highly prone to undergoing martensitic transformation at higher temperatures, above 500 °C, with the reduction in the M_s_ value at around 135 MPa and an increase in the pseudo-elastic region, ranging to 6%, as compared to TO500. XRD analyses (in Figure 4) reveal the coexistence of anatase TiO_2_ in a tetragonal structure and the presence of a hexagonal Ni_3_Ti phase. This discovery sheds light on the evolving crystallography of the material due to thermal oxidation. Further investigation into pseudo-elasticity uncovers a distinct trait, with the pseudo-elasticity measured at 6%, showcasing a noticeable increase in the plateau region. This increase is linked to the existence of hexagonal Ni_3_Ti, a phase that differs fundamentally in structure from the initial cubic B2 structure. The lack of coherence between hexagonal Ni_3_Ti and the B2 structure highlights the intricate transformations taking place within the wire [23].

Considering the SA and SASI variants, the wires exhibit behavior in correlation with REF wires, and this can be attributed to the fact that no temperatures above 100 °C have been used in these treatment processes. It is important to highlight that the fundamental piranha oxidation process mainly depends on chemical reactions rather than temperature-induced alterations, distinguishing it from other methods where temperature is the primary catalyst for oxidation activation. Although XRD identified traces of TiO_2_ in these samples, the oxide layer has less impact on the mechanical properties of these wires, and this proves the relatively lower impact of lower temperature treatments on the original properties of NiTi wires.

Considering the PO and PT of NiTi wires, the M_s_ transformation is almost not clearly detectable from the stress–strain relationship. In the range of 10–15% strain, at around 300 MPa of M_s_, it is presumed to have a short-range transformation plateau, and beyond that, the material undergoes plastic deformation. This behavior could be attributed to the plasma power and temperature of 380 °C that is reached naturally during the plasma process. This likely relieves internal stress in the samples [26], affecting the stress-induced martensitic transformation behavior. XRD analysis of the PT samples reveals a notable alteration in crystal structure, indicating the presence of nickel rich rhombohedral Ni_4_Ti. The phenomenon of gradual stress increase is attributed to localized stress variations around Ni_4_Ti_3_ precipitates within the material. This stress heterogeneity leads to sequential transformations: First, the B2-R transformation occurs, followed by R-B19′, primarily in regions with higher stress near Ni_4_Ti_3_ precipitates [27,28]. Simultaneously, a third transformation of R-B19′ occurs [23] in regions of lower stress, further from the Ni_4_Ti_3_ precipitates. The interplay of these transformations contributes to the observed gradual stress response [29,30].

**Figure 6:**
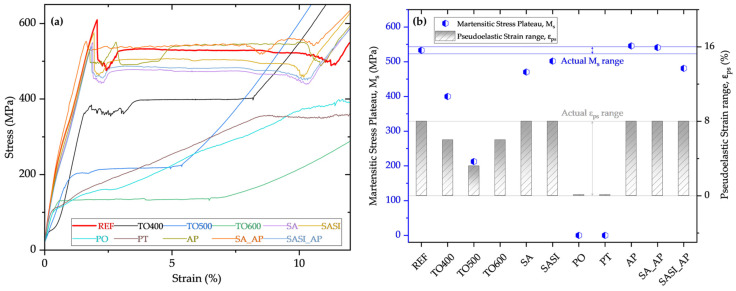
Plots depicting the (**a**) enlarged view of the M_s_ transformation plateau region vs. pseudo-elastic strain region and (**b**) the variation of the respective M_s_ and pseudo-elastic strain range values in all the treated and non-treated reference NiTi wires.

In the case of adhesion promoter-coated wires (AP, SA_AP, and SASI_AP), the M_s_ transformation occurs at around 500–550 MPa. The slight drop or deviations could be accounted for by the thick coating of AP on the circumference of the NiTi wires. On average, a significant portion of samples from all three treated wires show a martensitic elongation of approximately 8%, indicating consistent behavior in response to the adhesion promoter coatings. Interestingly, there are no observed alterations in the NiTi microstructure. An enlarged view of the M_s_ plateau stress ranges together with the pseudo-elastic strain range for all the wires is plotted in Figure 6.

**Figure 7:**
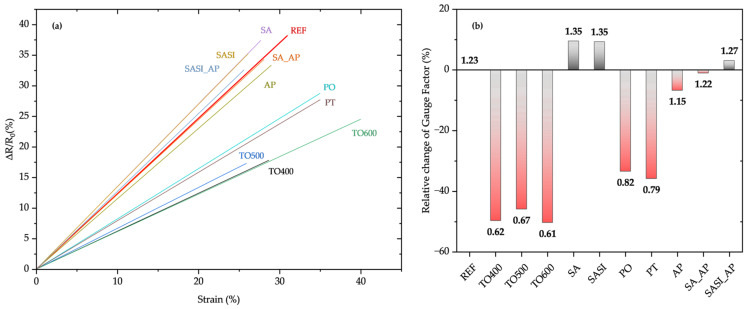
(**a**) The piezoresistive strain sensing response of non-treated and treated NiTi wires through in situ single-wire tensile testing. For comparison purposes, the linear fit data are plotted; (**b**) relative change of gauge factor values (decreasing values are denoted in red bars as compared to increasing values denoted in grey bars).

In addition to assessing mechanical strength, in situ electrical resistance measurements were collected from each of the single wires, and the data were linearly fitted to provide a comparative analysis. The NiTi wires, irrespective of the surface treatment methodologies, exhibit a linear response to the applied strain, as shown in Figure 7a.

The electrical response, as a correlation between the surface and mechanical analyses, is also influenced by the different surface treatment methodologies. The relative changes in gauge factor (GF) values are presented in Figure 7b. These results clearly demonstrate the degradation of strain sensitivity of the sensor wires with thermal and plasma-based surface treatments. Compared to untreated (REF) wires, thermally treated and plasma-treated wires show a GF degradation of approximately 50% and 40%, respectively. In contrast, chemically treated wires exhibit a slight increase of around 10% in gauge factor values, likely due to the removal of impurities or low-temperature thermal effects during the chemical treatment process. While adhesion promoter coatings, when used separately, result in a degradation of about 5% in GF values, combining these AP coatings with chemical treatments like SA and SASI helps regain the GF values close to the GF values of non-treated ones.

#### 3.3.2. Single-Wire Pullout Test Analyses

**Figure 8:**
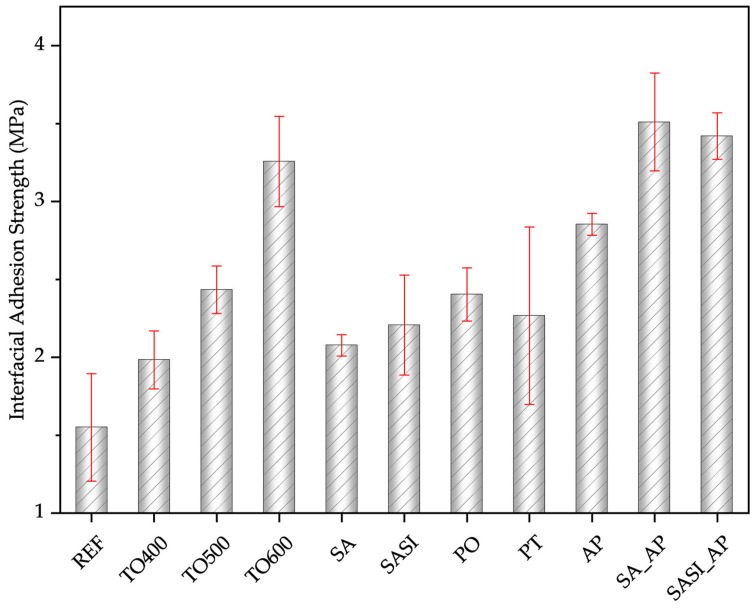
The interfacial adhesion strength of non-treated reference and treated NiTi wires.

To determine the influence of the different surface treatments carried out on the wires’ interfacial adhesion strength when being integrated into GFRP composite laminate structures, single-wire pull-out tests were carried out, and the plot can be seen in Figure 8.

Thermal oxidation treatments (TO400, TO500, and TO600) on NiTi wires exhibited increasing adhesion strength with rising oxidation temperatures. SEM analysis showed changes in surface morphology, including increased surface roughness, and altered pore characteristics, contributing to the enhanced adhesion strength. The highest adhesion strength among the TO treatments was achieved with TO600, indicating the positive effect of higher oxidation temperatures on adhesion. The adhesion strength increased by 28% for TO400, 61% for TO500, and 111% for TO600. However, it is to be noted that there is a compensation of the mechanical behavior of the NiTi wires at higher oxidation temperatures as discussed in Section 3.3.1.

Surface activation (SA) and surface-activated silane-coated (SASI) NiTi also resulted in a notable increase in adhesion strength due to the formation of hydroxide groups and silicone–oxygen bonds on the wire surface, respectively. However, the effectiveness is highly limited due to the presence of uneven pore distribution. The adhesion strength increased by 33% for SA and 41% for SASI.

Surface treatments such as plasma treatment (PT) and plasma oxidation (PO) also contributed to the enhanced adhesion strength, although less significantly compared to other treatments. SEM analysis of the plasma-treated wire surface revealed surface damage, indicating the importance of controlling surface roughness within a certain threshold to achieve the required adhesion strength. The adhesion strength increased by 45% for PT and 49% for PO. Here also it is to be noted that a negative mechanical property compensation should be crucial when achieving increasing interfacial adhesion strength.

The introduction of an adhesion promoter (AP) significantly enhanced the adhesion strength even without any pre-treatments, demonstrating the crucial role of the modified co-polyamide adhesion promoter in bolstering the bonding interface. The interfacial adhesion strength is improved by approx. 81% when non-treated wires are coated with an adhesion promoter (AP). Combining surface activation with an adhesion promoter (SA_AP) resulted in further enhanced adhesion strength, indicating a synergistic effect between these treatments. Surface-activated silane-coated NiTi (SASI) demonstrated increased adhesion strength due to the formation of silicone–oxygen bonds and chemical bonding with polyamide matrix. Adhesion strength increased by 127% for SAAP and 118% for SASI_AP.

Correlating this interfacial adhesion strength to the measured surface roughness, it is evident that the increasing surface roughness contributes to the increasing interfacial adhesion strength of the thermally treated wires by mechanical interlocking behavior. However, when the chemically treated wires (SA and SASI) and plasma-treated wires (PO and PT) are taken into consideration, the adhesion strength decreases irrespective of the increase in surface roughness. This could be attributed to the hypothesis that aggressive chemical and plasma reactions on the surface lead to the material softening and losing its mechanical capability to hold to the polymeric matrix strongly. By adding an adhesion promoter coating (AP, SA_AP, and SASI_AP), the adhesion strength is further improved, depicting the effectiveness of the co-polyamide coatings in the hybrid metal–plastic joining process.

## 4. Conclusions

This research study has successfully evaluated the impacts of various surface treatments on the NiTi material behavior and interfacial adhesion strength when integrated into GFRP using the hot-press process, and it has yielded significant insights. Thermal oxidation treatments at different temperatures showed a correlation between higher oxidation temperatures and stronger bonding, attributed to increased surface roughness and mechanical interlocking with the polyamide matrix of GFRP. Surface activation and adhesion promoter coatings demonstrated positive effects on adhesion strength individually, with a synergistic enhancement observed when combined. Silane coating also proved effective in enhancing adhesion strength, particularly when combined with surface activation and an adhesion promoter coating. Although plasma treatments showed improved interfacial adhesion to polyamide matrix, this happened only at the expense of mechanical properties, warranting further optimization. As this is ongoing research with further experimental investigations in progress to develop smart hybrid laminate structures with the optimal integration of SMA wires, future results detailing the integration mechanisms of NiTi sensors and their operational functional testing will be published soon.

## Figures and Tables

**Table 1:** Summary of experimental materials used and relevant sample naming details.

Sample ID	Surface Treatment Type	Materials Used	Temperature
-	-	-	(°C)
**REF**	Non-treated reference	-	-
**TO400**	Thermal treatment	-	400
**TO500**		-	500
**TO600**		-	600
**SA**	Chemical treatment	25% NH_4_OH; 30% H_2_O_2_; 5M NaOH	60
**SASI**		GPTMS; Xylene	120
**PO**	Plasma treatment	-	380
**PT**		-	380
**AP**	Adhesion Promoter coating	VESTAMELT^®^ Hylink copolyamide	200
**SA_AP**			200
**SASI_AP**			200

## Data Availability

The data that support the findings of this study are available from the corresponding author upon reasonable request.
